# Expanded utility of the R package, qgg, with applications within genomic medicine

**DOI:** 10.1093/bioinformatics/btad656

**Published:** 2023-10-26

**Authors:** Palle Duun Rohde, Izel Fourie Sørensen, Peter Sørensen

**Affiliations:** Genomic Medicine, Department of Health Science and Technology, Aalborg University, 9260 Gistrup, Denmark; Center for Quantitative Genetics and Genomics, Aarhus University, 8000 Aarhus, Denmark; Center for Quantitative Genetics and Genomics, Aarhus University, 8000 Aarhus, Denmark

## Abstract

**Summary:**

Here, we present an expanded utility of the R package qgg for genetic analyses of complex traits and diseases. One of the major updates of the package is, that it now includes Bayesian linear regression modeling procedures, which provide a unified framework for mapping of genetic variants, estimation of heritability and genomic prediction from either individual level data or from genome-wide association study summary data. With this release, the qgg package now provides a wealth of the commonly used methods in analysis of complex traits and diseases, without the need to switch between software and data formats.

**Availability and implementation:**

The methodologies are implemented in the publicly available R software package, qgg, using fast and memory efficient algorithms in C++ and is available on CRAN or as a developer version at our GitHub page (https://github.com/psoerensen/qgg). Notes on the implemented statistical genetic models, tutorials and example scripts are available at our GitHub page https://psoerensen.github.io/qgg/.

## 1 Introduction

With the increasing availability of large genetic biobanks and a simultaneous increase in number of collected samples by biobanks, there is a demand for software tools that allow for robust and efficient genomic analyses. Tools such as PLINK (Chang *et al.* 2015), GCTA (Yang *et al.* 2011), LDpred (Vilhjálmsson *et al.* 2015, Privé *et al.* 2021), LDAK (Speed and Balding 2014, Speed *et al.* 2017, Zhang *et al.*, 2021), and PRSice (Euesden *et al.* 2015) have changed the way researchers around the globe conduct genetic analyses of human complex traits and diseases. Here we present a major update of the R package qgg (Rohde *et al.* 2020), which has now been expanded to include a range of commonly used methods such as LD Score Regression (LDSC), adjustment of marker effect using correlated trait information (Rohde *et al.* 2022), a range of different Bayesian shrinkage models for gene mapping (Shrestha *et al.* 2023), and construction of single- and multiple trait polygenic scores (PGS). With a user-friendly interface, qgg offers a unified tool for quantitative genetic analysis of complex traits and diseases. Supplementary Tables S1–S4 contains an overview of the main functionalities that are implemented in the qgg package.

Human complex traits and diseases vary greatly in how their genetic architecture is shaped, e.g. in terms of effect size distribution, number of causal variants and non-linear genetic effects (Timpson *et al.* 2018). It is therefore important that the statistical model used for gene mapping or genomic risk prediction account for different types of genetic architectures. Bayesian linear regression (BLR) models have been proposed as a unified framework for gene mapping, estimation of genetic parameters and genomic prediction (Ehsani *et al.* 2012, 2016, Moser *et al.* 2015, Sørensen *et al.* 2015, Vilhjálmsson *et al.* 2015, Lloyd-Jones *et al.* 2019). BLR models account for the underlying genetic architecture by allowing the true genetic signal to be heterogeneously distributed over the genome (i.e. some regions have stronger genetic signal than others). Since BLR models fit all genetic markers simultaneously and account for linkage disequilibrium (LD) between markers, they often have greater power to detect causal associations and find less false negatives (Lloyd-Jones *et al.* 2019). The gain in statistical power to detect causal genetic variants subsequently increases the accuracy of genomic prediction.

## 2 Implementation

The software package qgg is available as an R package with main functions written in C++ taking advantage of fast and memory efficient algorithms. qgg handles large-scale data using efficient algorithms and by taking advantage of multi-core processing using openMP, multithreaded matrix operations implemented in BLAS libraries (e.g. OpenBLAS, ATLAS or MKL), and direct fast and memory-efficient processing of genetic data without the need to re-format the binary PLINK files (Chang *et al.* 2015), as is needed in other R packages for handling large-scale genetic data.

## 3 Usage

**Figure 1.**
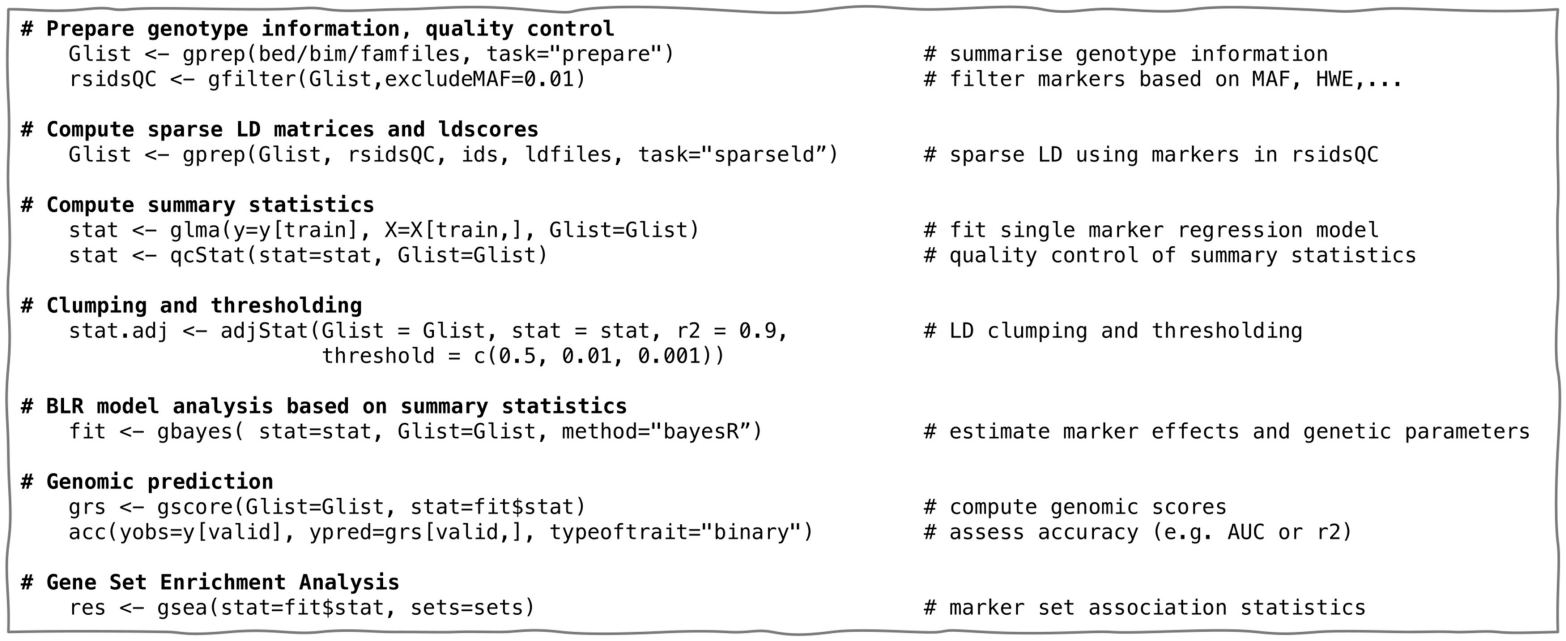
Overview of the simple and streamlined workflow for genetic analysis of complex traits that takes PLINK files as input. The output from each function is designed to match the input format of downstream analyses.

The different statistical genetic methods are implemented using a simple and streamlined workflow (Fig. 1). Initially, PLINK files are processed to construct a Glist-object that summarizes information about the genetic data and computes genotype and allele frequencies, genotype missingness etc. This information can be used to perform standard quality control of the genetic data and to compute sparse LD matrices and LD scores. After the initial pre-processing, genome-wide association study (GWAS) summary data can be directly computed, followed by adjustment using either clumping and thresholding (C+T) or one of the implemented BLR models (bayes A, bayes C, bayes R, bayesian Lasso and bayesian mixed model). Finally, the GWAS summary data can be used to construct PGS or to identify biologically relevant gene sets enriched for associated variants.

In the accompanying Supplementary Material we showcase the new features implemented in qgg, by performing single- and multi-trait genetic analyses of body mass index (BMI) and standing height using data from the United Kingdom Biobank (UKB) (Bycroft *et al.* 2018).

## 4 Conclusion

We have presented an update of the R package qgg. We highlight the BLR models for single and multiple trait analyses for (i) constructing PGS with utilities in genomic medicine, (ii) fine mapping of GWAS results and (iii) accurate estimation of quantitative genetic parameters.

## Supplementary Material

btad656_Supplementary_Data

## Data Availability

The phenotypic and genotype data underlying this article cannot be shared publicly. The data comes from the UK Biobank, which researchers can gain access to by applying for research access. See https://www.ukbiobank.ac.uk/enable-your-research/apply-for-access for instructions for the application process.
