# Supplementary material for: Expanded utility of the R package, qgg, with applications within genomic medicine
Source: Bioinformatics. 2023 Oct 26;39(11):btad656. doi: 10.1093/bioinformatics/btad656 (PMC10627350; doi:10.1093/bioinformatics/btad656)
Supplement: btad656_Supplementary_Data [file btad656_supplementary_data.pdf]

Supplementary Material

Expanded utility of the R package, qgg, with applications within genomic medicine

Palle Duun Rohde[1], Izel Fourie Sørensen[2], and Peter Sørensen[2]

[1]Genomic Medicine, Department of Health Science and Technology, Aalborg University, Aalborg, Denmark,
[2]Center for Quantitative Genetics and Genomics, Aarhus University, Aarhus, Denmark

S1 Introduction

This supplementary document contains supporting material for the manuscript "*Expanded utility of the R package, qgg, with applications within genomic medicine*". First some details on the different statistical genetic models are described. Then, we showcase some of the new features by genetic analyses of human height and body mass index (BMI) utilising genetic and phenotypic data from the UK Biobank (UKB) (Bycroft *et al.*, 2018).

S2 Overview

In the following we provide an overview of the most commonly used functions and arguments in the `qgg` package.

- Table S1: Processing of genetic data, computation of LD, and quality control (QC) of genetic data and GWAS summary statistics.
- Table S2: Functions and arguments related to single and multiple genetic variant associations.
- Table S3: Functions and arguments related to the implemented Bayesian Linear Regression (BLR) models..
- Table S4: Functions and arguments related to construction of polygenic score (PGS).

Table S1: Processing of genetic data, computation of LD, and quality control (QC) of genetic data and GWAS summary statistics. Consult the package documentation for complete description of all functions and arguments.

FUNCTION	ARGUMENTS	DESCRIPTION
`gprep()`	`task="prepare"`	Reads binary PLINK files (in the format *.bed, *.bim, *.fam), and stores basic information on the genetic variants such as alles (A1 and A2), number of carriers, and computes simple summary statistics for the genotype data such as allele frequencies, missingness, homozygosity and heterozygosity and store this information in a user-defined R-object, `Glist`.
	`task="sparseld"`	Computes sparse LD matrix using genotypes in `Glist` for a subset of variants specified by `rsids=NULL` based on a window size given by `msize=NULL`.
	`task="ldscores"`	Computes LD scores (i.e., squared sum of LD/correlation (i.e., r^2) between markers) based on the sparse LD matrix (which is prepared under `task="sparseld"`) information in `Glist`.
`getG()`	`scale=TRUE`	Get genotypes for a pre-specified chromosome (`chr=NULL`) for a subset of genetic variants (`rsids=NULL`) for a subset of individuals (can be specified with `ids=NULL`) in `Glist`. Genotypes can be represented as allele counts (`scale=FALSE`, coded as 0, 1, 2 counting the number of A1-alleles, the first allele listed in the *.bim file) or centered and scaled (`scale=TRUE`).
`gfilter()`	`Glist=Glist`	Perform standard quality control on the genotypes in `Glist`.
`checkStat()`	`stat=stat`	Perform standard quality control on GWAS summary statistics (`stat`) based on genotypes in `Glist`.

Table S2: Functions and arguments related to single and multiple genetic variant associations. Consult the package documentation for complete description of all functions and arguments.

FUNCTION	ARGUMENTS	DESCRIPTION
`glma()`		Performs linear regression between a response variable and genetic variants in `Glist` or a subset of variants stored in a matrix (obtained with `getG()`). Covariates for adjusting the quantitative response can be given in a design matrix (`X=NULL`).
`grm()`	`Glist=Glist`	Computes the genomic relationship matrix using genotypes in `Glist`, save the GRM in a binary file on the disk (specified by `fnG=NULL`) and store in a user-defined R-object, `GRMlist`.
	`W=W`	Computes genomic relationship matrix using genotypes from a centred and scaled n-by-m genotype matrix (obtained using `getG()`).
`greml()`	`GRMlist=GRMlist`	Estimate variance components based on Genomic Restricted Maximum Likelilood and using GRMlist stored on disk.
	`GRM=GRM`	Using GRM in memory.
`gsea()`	`method="hyperg"`	Performs gene set enrichment analysis (GSEA) utilising a hypergeometric test.
	`method="sum"`	Performs a summation-based GSEA.
	`method="score"`	A score-based GSEA similar to what is implemented in e.g., SKAT (Wu *et al.*, 2011).
	`method="cvat"`	Performs the Covariance Association Test (CVAT) (Rohde *et al.*, 2016).
`ldsc()`	`what="h2"`	Compute the narrow-sense heritability (h^2) using linkage disequilibrium (LD) score regression based on pre-computed LD scores from `Glist` and GWAS summary statistics.
	`what="rg"`	Compute the genetic correlation between pairs of traits using LD score regression.

Table S3: Functions and arguments related to the different Bayesian Linear Regression (BLR) models that are implemented. Consult the package documentation for complete description of all functions and arguments.

Function	Arguments	Description
`gbayes()`		The `gbayes`-function fit a Bayesian Linear Regression (BLR) on either individual level data (*i.e.*, a phenotype use argument `y=NULL`) and genotypes (use `Glist=NULL` or `W=NULL`), or GWAS summary data (use argument `stat=NULL`).
	`methods="bayesN"`	Fits a BLR model where the marker effects follows a normal distribution with a uniform variance across all markers.
	`methods="bayesA"`	Incorporates prior knowledge on marker variance using an inverted chi-square distribution.
	`methods="bayesL"`	Fits a Bayesian Lasso model where none markers receive zero-effect, but some are assigned a very small value.
	`methods="bayesC"`	Markers are assigned to one of two groups; non-zero effect or zero effect.
	`methods="bayesR"`	Markers are assigned to one of four groups; three non-zero effect groups or zero effect.
`gmap()`		Performs fine mapping using one of the implemented BLR methods.

Table S4: Functions and arguments related to construction of polygenic scores (PGS). Consult the package documentation for complete description of all functions and arguments.

FUNCTION	ARGUMENTS	DESCRIPTION
`adjStat()`		Adjust marker summary statistics based on clumping using an LD cutoff (r^2, `r2=NULL`) and a P-value threshold (`threshold=NULL`)
`gscore()`		Computes polygenic scores (PGS) using single marker summary statistics and genotypes in Glist.
`mtAdj()`		Adjust marker effects using correlated trait information (e.g. genomic correlation obtained using `ldsc()`)).
`acc()`		Compute prediction accuracies of the PGS. If argument `typeoftrait="binary"` Nagelkerks variance explained and AUC is reported.

S3 Methods

S3.1 Bayesian linear regression models

Complex traits and multifactorial diseases are likely to be highly polygenic, with hundreds to thousands of causal variants that have small effect sizes (Manolio *et al.*, 2009; Timpson *et al.*, 2018). Bayesian linear regression (BLR) models provide a unified framework for gene mapping, prediction of genetic predisposition, estimation of genetic parameters, and effect size distribution (Moser *et al.*, 2015; Lloyd-Jones *et al.*, 2019; Patxot *et al.*, 2021). BLR models use many linked markers to jointly estimate marker effects and account for the underlying genetic architecture of the trait. This allows for a more accurate estimate of the true underlying genetic signal leading to better predictions. BLR models can also map genetic variants associated with phenotypes, estimate the total variance explained by the genetic markers, and provide information about the genetic architecture of the trait. To match the true distribution of marker effects, a mixture of normal distributions is used as a prior for the effect sizes. Zero variance or a mixture of normal distributions with varying variances can be included in the prior. Extensions for handling multiple marker sets and multiple traits for the BLR models are also presented. Overall, BLR models provide a powerful tool for understanding the genetic basis of complex traits and multifactorial diseases.

Statistical model The multiple linear regression model relates the phenotype to a set of genetic markers. The model can be written as:

$$\mathbf{y} = \mathbf{X}\mathbf{b} + \mathbf{e},$$

where $\mathbf{y}$ is a vector of standardised phenotypic observations, $\mathbf{X}$ is a matrix of SNP genotypes where columns are standardised to zero mean and variance 1, $\mathbf{b}$ is a vector of genetic effects for each SNP, and $\mathbf{e}$ is a vector of residual errors. The dimensions of $\mathbf{y}$, $\mathbf{X}$, $\mathbf{b}$, and $\mathbf{e}$ depend on the number of traits, k, the number of SNP markers, m, and the number of individuals, n. The residuals, $\mathbf{e}$, are assumed to be independently and identically distributed multivariate normal with a mean of zero and a covariance matrix of $\mathbf{I}\sigma_e^2$, where σ_e^2 is the variance of the residual errors.

Estimation of parameters using Bayesian methods In the Bayesian multiple regression model, the posterior density of the model parameters (b,σ_b^2,σ_e^2) depends on the likelihood of the data given the parameters and a prior probability for the model parameters:

$$p(b, \sigma_b^2, \sigma_e^2|y) \propto p(y|b, \sigma_b^2, \sigma_e^2)p(b|\sigma_b^2)p(\sigma_b^2)p(\sigma_e^2)$$

The prior density of marker effects, $p(b|\sigma_b^2)$, determines whether the model will induce variable selection and shrinkage or shrinkage only. The choice of prior also determines the extent and type of shrinkage induced. Ideally, the choice of prior for the marker effect should reflect the genetic architecture of the trait and may vary significantly across traits. Most complex traits and diseases are likely to be highly polygenic, with hundreds to thousands of causal variants, most frequently of small effect. Therefore, the prior distribution must include many small and few large effects. Additionally, marker effects are a priori assumed to be uncorrelated, although markers can be in strong linkage disequilibrium and therefore have a high posterior correlation. Many priors for marker effects have been proposed, but they often come more from practical considerations (such as ease of computation) than biological reasons. Each prior gives rise to a method or family of methods, and we will describe some of them next, along with their implications.

Prior marker variance BayesN In the BayesN approach, the prior of the marker effect, b, follows a priori a normal distribution with a variance σ_b^2 which is constant across markers:

$$p(b) = \prod_i p(b_i)$$

where

$$p(b_i) = N\left(0, \sigma_b^2\right)$$

The normal distribution typically has the majority of effects clustered around 0, with only a small number of effects being considerably large. This means that assuming normality as a prior probability distribution may prevent markers with large effects from being identified, unless there is significant additional information available to offset this prior assumption (Meuwissen *et al.*, 2001).

Prior marker variance BayesL In the BayesL approach, marker effects are not set to zero, but some are assigned with a very small value that follows the prior distribution (de Los Campos *et al.*, 2009; Park and Casella, 2008; Legarra *et al.*, 2011):

$$p(b_i|\sigma_{b_i}^2) = N(0, \sigma_{b_i}^2),$$

where the variance is a Laplace variance distribution,

$$p(\sigma_{b_i}^2|\lambda) = \frac{\lambda^2}{2}\exp(\frac{\lambda^2\sigma_{b_i}^2}{2\sigma_e^2}).$$

The prior value of λ can be expressed as the ratio between marker variance and residual variance (Pérez *et al.*, 2010):

$$\frac{\lambda^2}{2} = \frac{\sigma_g^2}{\sigma_e^2 2\sum_i p_i(1-p_i)},$$

where σ_g^2 is the total additive genetic variance and p_i represents the allele frequency of the i-th marker.

Prior marker variance BayesA In the BayesA approach, it is assumed that we have prior knowledge of the marker variance, denoted as σ_b^2. This value is used to inform the prior distribution for $\sigma_{b_i}^2$ using an inverted chi-squared distribution with υ_b degrees of freedom and scale parameter $S_b^2 = \upsilon_b\sigma_b^2$. The prior distribution for the marker effects is then a normal distribution with mean 0 and variance $\sigma_{b_i}^2$.

In the second stage, we assume a prior distribution for the marker variances themselves using the inverted chi-squared distribution with parameters υ_b and S_b^2. The value of σ_b^2 is set to $\frac{(\upsilon_b-2)}{\upsilon_b}\frac{\sigma_g^2}{2\sum_i p_i(1-p_i)}$, where σ_g^2 is the genetic variance and p_i is the frequency of the effect allele at the ith marker. This corresponds to a prior on the marker effects that follows a scaled t distribution with mean 0 and υ_b degrees of freedom, and has fatter tails than a normal distribution (Gianola *et al.*, 2009).

Overall, the BayesA approach incorporates prior knowledge about the marker variance and produces a prior distribution for marker effects that is more likely to include large effects compared to a normal distribution.

Prior marker variance BayesC In the BayesC approach, the marker effects ($\mathbf{b}$) are assumed to follow a mixture distribution with a point mass at zero and a univariate normal distribution with a common marker effect variance σ_b^2. This reflects the common belief that only a few loci have a causal effect on the trait. To implement this, an additional variables δ_i is introduced that indicate whether the i-th marker has an effect or not (Habier *et al.*, 2011). These variables δ have a prior distribution called Bernoulli with a probability π of being zero. Thus, the prior hierarchy is:

$$p(b_i|\sigma_b^2, \pi) = \begin{cases} 0 & \text{with probability } \pi \\ \sim N(0, \sigma_b^2) & \text{with probability } 1-\pi, \end{cases}$$

The prior distribution for the marker variances $\sigma_{b_i}^2$ is an inverted chi-squared distribution with parameters υ_b and $S_b^2 = \sigma_b^2\upsilon_b$, where $\sigma_b^2 = \frac{\sigma_g^2}{(1-\pi)2\sum_i p_i(1-p_i)}$ and σ_g^2 is the genetic variance. This choice of

prior reflects the assumption that the majority of markers have no effect, and that the effect sizes of the remaining markers are drawn from a distribution with heavy tails.

Overall, the BayesC approach introduces a prior distribution that favours sparse models with a few markers having large effects, while the majority of markers have no effect. The prior distribution used in BayesC is similar to what is implemented in the commonly used method LDpred (Privé *et al.*, 2021; Vilhjálmsson *et al.*, 2015).

Prior marker variance BayesR In the Bayes R approach, the marker effects, denoted **b**, are assumed to come from a mixture distribution consisting of a point mass at zero and univariate normal distributions conditional on a common marker effect variance σ_b^2 and variance scaling factors γ (Lloyd-Jones *et al.*, 2019; Erbe *et al.*, 2012; Moser *et al.*, 2015). This is expressed as follows:

$$p(b_i|\sigma_b^2, \pi) = \begin{cases} 0 & \text{with probability } \pi_1 \\ \sim N(0, \gamma_2\sigma_b^2) & \text{with probability } \pi_2 \\ \vdots & \vdots \\ \sim N(0, \gamma_C\sigma_b^2) & \text{with probability } 1 - \sum_{C=1}^{C-1} \pi_C, \end{cases}$$

where $\pi = (\pi_1, \pi_2, \ldots, \pi_C)$ is a vector of prior probabilities and $\gamma = (\gamma_1, \gamma_2, \ldots, \gamma_C)x$ is a vector of variance scaling factors for each of the C marker variance classes. The γ scalars are predefined and constrain how the marker effect variance, σ_b^2, scales within each class distribution. Typically, four classes are used (Lloyd-Jones *et al.*, 2019) $\gamma = (0, 0.01, 0.1, 1.0)$ with the probabilities $\pi = (0.95, 0.02, 0.02, 0.01)$.

The prior distribution for the marker variance, σ_b^2, is assumed to be an inverse chi-square distribution, $\chi^{-1}(S_b, \nu_b)$.

The proportion of markers in each mixture class, π, follows a Dirichlet $(C, c + \alpha)$ distribution, where c is a vector of length C that contains the counts of the number of variants in each variance class and $\alpha = (1, 1, 1, 1)'$.

To introduce the concept of data augmentation, an indicator variable $d = (d_1, d_2, \ldots, d_{m-1}, d_m)$ is used, where d_j indicates whether the jth marker effect is zero or non-zero.

Estimation of model parameters BLR methods use an iterative algorithm for jointly estimating genetic marker effects. Estimation of the joint marker effects depends on additional model parameters such as the probability of being causal (π), an overall marker variance (σ_b^2), genetic variance (σ_g^2) and residual variance (σ_e^2), which can be used for estimating trait heritability, $h_{SNP}^2 = \frac{\sigma_g^2}{\sigma_g^2 + \sigma_e^2}$. Estimation of the model parameters is obtained using Markov chain Monte Carlo (MCMC) Gibbs sampling from the fully conditional posterior distributions (Habier *et al.*, 2011; Legarra *et al.*, 2011; Lloyd-Jones *et al.*, 2019).

To illustrate the concept, consider the following MCMC algorithm used to obtain estimates of the parameters in the BayesR models (Lloyd-Jones *et al.*, 2019). The multiple linear regression model is parameterised in terms of $\theta = (\sigma_e^2, \sigma_\beta^2, \gamma, \pi, b, d)$. The full conditional sampling distributions for these parameters are presented below.

The joint posterior for all parameter in the multiple regression model can be written as:

$$f\left(\sigma_e^2, \sigma_\beta^2, \gamma, \pi,\ b,\ d|y\right) \propto \left(y|\sigma_e^2, \sigma_\beta^2, \gamma, \pi,\ b,\ d\right) f\left(b|d, \gamma, \sigma_\beta^2\right) f\left(d|\pi\right) f\left(\sigma_e^2\right) f\left(\sigma_\beta^2\right) f\left(\pi\right)$$

The parameters d_j and b_j are sampled jointly from their joint full conditional distributions, which can be written as the product of the full conditional distribution of b_j given d_j and the marginal full conditional distribution of d_j:

$$f_j\left(d_j, b_j|\theta_{-j}, \widetilde{\mathbf{y}}\right) = f_j\left(b_j|\theta_{-j}, d_j, \widetilde{\mathbf{y}}\right) f_j\left(d_j, |\theta_{-j}, \widetilde{\mathbf{y}}\right),$$

where θ_{-j} is all parameters except d_j and b_j, and $\widetilde{\mathbf{y}} = \mathbf{y} - \mathbf{X}_{-j}\mathbf{b}_{-j}$ is the phenotype adjusted for all marker effects except for j'th marker (b_j).

The full conditional distribution for b_j can be be written as:

$$f_j\left(b_j|\theta_{-j}, d_j, \widetilde{\mathbf{y}}\right) \propto N\left(C_j^{-1} r_j, C_j^{-1}\right),$$

where $C_j^{-1} = \frac{\sigma_e^2}{x_j^{'} x_j + \frac{\sigma_e^2}{\sigma_c^2}}$ and $r_j = \frac{x_j^{'}\widetilde{y}}{\sigma_e^2}$.

The marginal full conditional probability for the indicator variable of d_j is:

$$f_j\left(d_j = c|\theta_{-j}, \widetilde{\mathbf{y}}\right) = \frac{f_j\left(\widetilde{\mathbf{y}}|d_j = c, \theta_{-j}\right) f\left(d_j|\pi_c\right)}{\sum_{k=1}^{C} f_j\left(\widetilde{\mathbf{y}}|d_j = k, \theta_{-j}\right) f\left(d_j|\pi_k\right)},$$

where $f_j\left(\widetilde{y}|d_j = c, \theta_{-j}\right) = \left(\sigma_c^2\right)^{-0.5}\left(C_j^{-1}\right)^{0.5} \exp\left[\frac{1}{2} C_j^{-1} r_j^2\right]$.

The categorical distribution probabilities for any number of mixture components can be computed from the expressions mentioned above. These probabilities are then used to sample from a categorical distribution, which determines the class of the variant to be sampled. The effect of the variant is sampled from a relevant normal distribution or assigned a zero effect based on the marker variance class. To sample from the categorical distribution, a vector of cumulative probabilities is created in order of the categories, and the lowest value 'c' is accepted such that the cumulative probability is greater than a uniform distribution 'u', sampled from $U(0,1)$.

The proportion of markers in each mixture class π follows a Dirichlet distribution with parameters $C, c + \alpha$, where $\mathbf{c}$ is a vector containing the count of the number of variants in each variance class, α is a vector of ones, and C is the number of mixture components.

The full conditional posterior distribution for σ_b^2 is a scaled inverse chi-squared distribution with $v_b = \widetilde{\nu}_b + q$ degrees of freedom and scale parameter $S_b^2 = \frac{\widetilde{\nu}_b \widetilde{S}_b^2 + \sum_{j=1}^{q} \frac{b_j^2}{\gamma_{d_j}}}{\widetilde{\nu}_b + q}$, where $\widetilde{\nu}_b$ and $\widetilde{S}_b^2$ are the prior degrees of freedom and scale parameters, and q is the number of predictors.

The full conditional posterior distribution for σ_e^2 is a scaled inverse chi-squared distribution with $v_e = \widetilde{\nu}_e + n$ degrees of freedom and scale parameter $S_e^2 = \frac{\widetilde{\nu}_e \widetilde{S}_e^2 + SS_e}{\widetilde{\nu}_e + n}$, where $\widetilde{\nu}_e$ and $\widetilde{S}_e^2$ are the prior degrees of freedom and scale parameters, and $SS_e = \mathbf{y}^{'}\mathbf{y} - \mathbf{b}^{'} r^{*} - \mathbf{b}^{'}\mathbf{X}^{'}\mathbf{y}$, where $\mathbf{y}$ is the vector of phenotypic observations, $\mathbf{X}$ is the design matrix, $\mathbf{b}$ is the effect size vector, and r^{*} is the sum of the marker effects.

Extension to summary statistics The multiple regression model depends on the marker effects as the main parameter of interest. In case genotypes are unavailable, $\mathbf{X}'\mathbf{y}$ and $\mathbf{X}'\mathbf{X}$ can be reconstructed from a linkage disequilibrium (LD) correlation matrix $\mathbf{B}$ using summary statistics:

$$\mathbf{X}'\mathbf{X} = D^{0.5}\mathbf{B}D^{0.5}$$

D_i is defined as $\frac{1}{\sigma_{b_i}^2 + b_i^2/n_i}$ if the genotypes are centered to mean 0, or $D_i = n_i$ if they are entered to mean 0 and scaled to unit variance. Furthermore,

$$\mathbf{X}'\mathbf{y} = D\mathbf{b}_m$$

where $\mathbf{b}_m = D^{-1}\mathbf{X}'\mathbf{y}$ represents the marginal marker effects obtained from a standard genome-wide association study. To construct the reference LD correlation matrix, $\mathbf{B}$, a fixed window approach of 1-10 Mb is typically used, with LD correlation values outside this window set to zero.

S3.2 Extension to multiple traits

Bayesian linear regression models can be extended to analyse multiple traits, which is useful for understanding the genetic correlations between traits using genetic markers. This approach can also help evaluate

whether pleiotropy or linkage disequilibrium explain between-trait associations, and can improve the accuracy of genomic predictions by leveraging pleiotropy among traits. One common approach to multiple trait analysis assumes that a locus simultaneously affects all the traits or none of them (Jia and Jannink, 2012). While this approach is simple, it may require estimating many effects for loci that have no effect on a trait, which can erode prediction accuracy. Additionally, this assumption is not biologically meaningful, especially for analyses involving many traits. To address this limitation, a general multiple trait Bayesian linear regression model based on the BayesC prior has been proposed (Cheng *et al.*, 2018). This model allows a locus to affect any combination of traits, providing insight into whether markers affect all, some, or none of the traits. For example, the model can estimate the proportion of markers in each of the (0,0), (1,0), (0,1), and (1,1) categories, where (0,0) means ”no effect” and (1,1) denotes ”effect” on both traits. However, this model can be computationally intensive for large numbers of traits. One way to reduce computational complexity is to assume the same correlation for all markers when using multivariate prior distributions for marker effects.

S3.3 Fine-mapping with BLR models

BLR models used for fine-mapping are specialized to identify markers that have the highest probability of being causal. For example, BLR models based on a mixture of normal priors (e.g., BayesC or BayesR) can be used to compute the posterior inclusion probability (PIP) of a marker, which is the proportion of iterations from the Gibbs sampling for which the marker is included in the model with a non-zero effect (Schaid *et al.*, 2018). Ranking markers by their PIP is a useful way to identify potential causal markers. For instance, the top k markers ranked by their PIP are expected to maximize the number of causal markers across all possible subsets of size k. However, if multiple markers in a region are highly correlated and all are approximately equally associated with the phenotype, it may be better to estimate the posterior expected number of causal markers by summing the estimated PIPs for all markers in that region. Thus, the sum of PIPs for m markers in a window can be used to make inferences about the presence of causal markers in that window. This statistic is known as the window posterior inclusion probability:

$$T_{PIP} = \sum_{i=1}^{m} PIP_i$$

A sliding genomic window size of 100 markers was used in this study.

S3.4 BLR-based gene set enrichment analyses

Aggregating genetic markers into biologically informed entities, such as genes, biological pathways, protein interaction complexes etc., and analysing those sets of markers jointly constitute a valuable addition to single-marker analyses (de Leeuw *et al.*, 2016; Rohde *et al.*, 2016; Sørensen *et al.*, 2017). The qgg package (Rohde *et al.*, 2020) contains a range of different ge0ne set enrichment analyses (GSEA), that use marginal OLS test statistics or P-values when the input data is GWAS summary statistics (in contrast to individual level genetic data). Here, we showcase the utility of using the sum of posterior inclusion probabilities from BayesC in gene sets defined by genes, gene ontologies, pathways, protein complexes and chemical complexes. In this study, genetic markers were mapped to 1) genes and gene ontologies (Ashburner *et al.*, 2000) using the Bioconductor package org.Hs.eg.db (Carlson, 2019), 2) biological pathways using data from the Reactome database (Gillespie, 2022), and 3) protein complexes and chemical complexes using data from STRING and STICH, respectively (Szklarczyk *et al.*, 2016, 2019).

S3.5 Polygenic Scores (PGS)

A polygenic score (PGS) captures an individual's genetic predisposition for a given multifactorial trait, and can be computed as (Dudbridge, 2013; Purcell *et al.*, 2009):

$$PGS = \sum_{i=1}^{m} \mathbf{X}_i \hat{b}_i,$$

where $\mathbf{X}_i$ denotes the i-th genotype (encoded as 0, 1, 2 counting the number of the alternative allele), and $\hat{b}_i$ is the estimated SNP effect for the i-th SNP on the phenotype (for binary outcomes then $\hat{b}_i = log(\hat{OR}_i)$, where $\hat{OR}$ is the estimated odds ratio). The marker effects ($\mathbf{b}$) are typically marginal effects originating from linear/logistic regressions (Chang *et al.*, 2015), linear mixed models (Yu *et al.*, 2006; Zhou and Stephens, 2012) or using BLRs models (Loh *et al.*, 2015; Mbatchou *et al.*, 2021). In absence of shrinkage of the marginal effects, several different approaches can be applied to select genetic variants used in constructing a PGS, which are described below.

S3.5.1 Clumping and thresholding

PGS's are commonly computed using the appraoch clumping and thresholding (C+T) (Euesden *et al.*, 2015), which involves sorting all genetic variants based on their association P-value statistics, following removal of variants that have a squared correlation coefficient (r^2) greater than a certain threshold (Choi *et al.*, 2020).

Important parameters to optimise C+T for polygenic scoring are r^2 and the P-value significance level (*e.g.*, Privé *et al.* (2019)). For different r^2- and P-thresholds the C+T predictors can be computed.

S3.6 Multi-trait PGS

We and others have previously shown how a multiple-trait (MT) genomic predictor can be created as a weighted index combining several GWAS summary statistics, thereby taking advantage of genetic correlations among traits and diseases (Maier *et al.*, 2018; Rohde *et al.*, 2021) . In short, the index weights are obtained as:

$$\mathbf{w} = \begin{bmatrix} \frac{h_1^2}{M} + \frac{1}{N_1} & \cdots & \frac{r_g h_1 h_k}{M} \\ \vdots & \ddots & \vdots \\ \frac{r_g h_k h_1}{M} & \cdots & \frac{h_k^2}{M} + \frac{1}{N_k} \end{bmatrix}^{-1} \begin{bmatrix} \frac{h_1^2}{M} \\ \vdots \\ \frac{r_g h_k h_1}{M} \end{bmatrix},$$

where h^2 and r_q denotes the estimated heritability and genetic correlations, respectively, M is the number of independent chromosomal segments (M=60,000 (Yang *et al.*, 2011), and N is the sample size from each individual GWAS. Here, the heritability and genetic correlations were estimated with LDSC as implemented in the qgg package. The multi-trait PGS can be obtained as the sum of adjusted marker effects ($\hat{\mathbf{b}}_{w_{MT_i}} = w_i^{'}\hat{\mathbf{b}}_i$):

$$MT - PGS = \sum_{i=1}^{m} \mathbf{X}_i \hat{\mathbf{b}}_{w_{MT_i}}$$

Further details on all the statistical genetic models presented in the Materials and Methods section, can be found at our accompanied homepage: www.qganalytics.com/qg-notes.

S3.7 Genetic and phenotypic data

Genetic and phenotypic data were obtained from the United Kingdom Biobank (UKB), in which data has been collected for more than 500.000 individuals aged 37-73 years (Bycroft *et al.*, 2018). Genotyping details has been described previously (Bycroft *et al.*, 2018). To obtain a genetic homogeneous study population we restricted our analyses to unrelated British, Caucasians and excluded individuals with more than 5,000

missing genotypes or individuals with autosomal aneuploidy, resulting in a total of n=335,744 samples. To present the novel utilities of the QGG package we used the genotyped variants and excluded those (1) with minor allele frequency < 0.01, (2) that deviate from Hardy-Weinberg equilibrium (P-value $< 1\times10^{-12}$), (3) where genetic variants were located within the major histocompatibility complex, (4) with allele ambiguous (i.e., GC or AT), (5) that were multi-allelic or an insertion/deletion (Marees *et al.*, 2018). This resulted in a total of 533,679 genotyped variants.

Standing height (data field 50) and BMI (data field 21001) were used as example traits. Prior to analyses the two traits were for each sex separately adjusted for age, UKB assessment centre and the first ten genetic principal components following inverse rank normalisation.

Linkage disequilibrium (LD) among quality-controlled genotype variants was computed using a random selection of 50,000 UKB participants in window sizes of 2000 variants on each side of a genotyped marker. LD was computed as the observed correlation among genotyped variants as $LD_x = W_x^{'}W_x/(n-1)$, where x was the index of the set of variants for which LD was computed, W was a centred and scaled (to mean zero and variance one) genotype matrix, and n was the number of individuals.

S4 Results

S4.1 Polygenic prediction of standing height and body mass index

Utilising phenotypic and genetic data from the UKB (Bycroft *et al.*, 2018), the white-British unrelated sub-cohort was split into five random training cohorts each containing 300,000 individuals. First OLS association tests between trait phenotype and quality-controlled genotype variants were performed for each sex separately, and combined, within each training cohort.

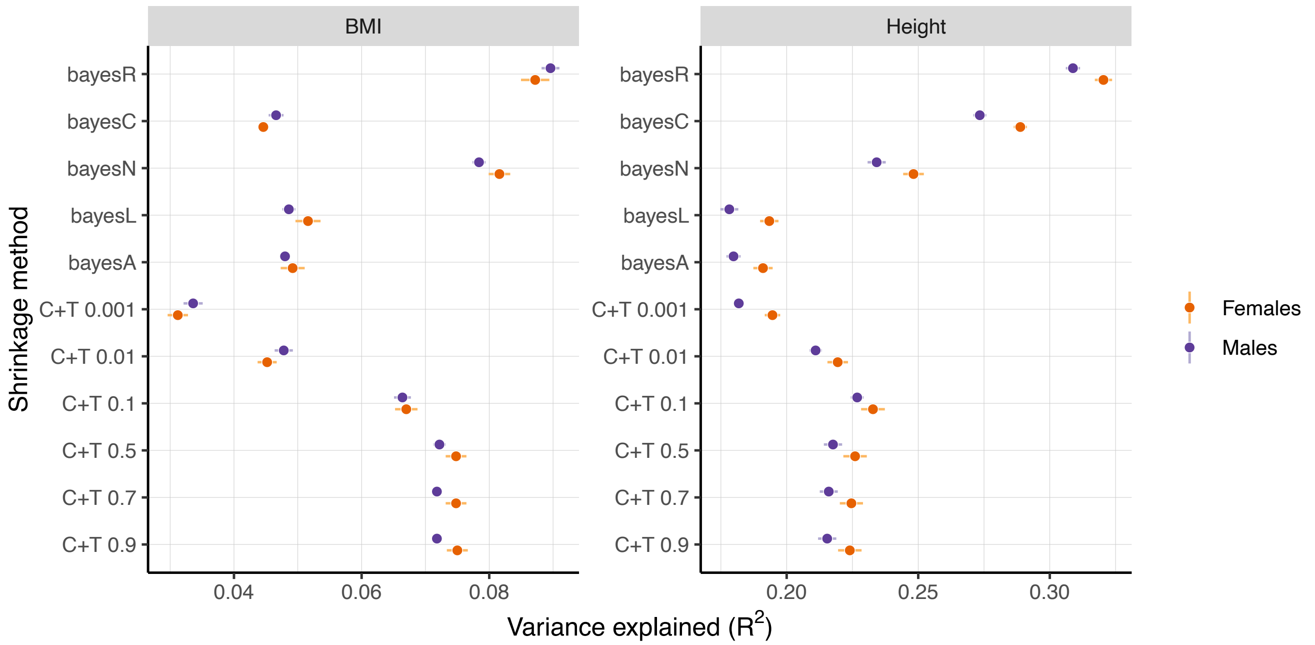

Figure S1: Average prediction accuracies (error bars are standard errors of the mean) for body mass index (BMI) and standing height for males and females separately, quantified as variance explained (R^2). Different shrinkage algorithms were tested including clumping and thresholding (C+T, P<(0.001,0.01,0.1,0.5,0.7,0.9)), and five different Bayesian Linear Regression (BLR) models.

The genetic marker effects ($\hat{\mathbf{b}}$) from each training cohort was used to construct sex-specific PGS for the individuals not included within the training cohorts. Expectedly, the P-value thresholds used in C+T showed different prediction accuracy optima for the two traits (Figure S1), and both BayesC and BayesR

outperformed the other Bayesian shrinkage models and C+T (Figure S1). No difference in predictive performance between sexes was seen for BMI (Figure S2), however, for standing height, the prediction accuracy was significantly higher for females compared with males when using the BLR models (Figure S2). Next, we computed multi-trait PGS by first constructing index selection weights. The MT-PGS

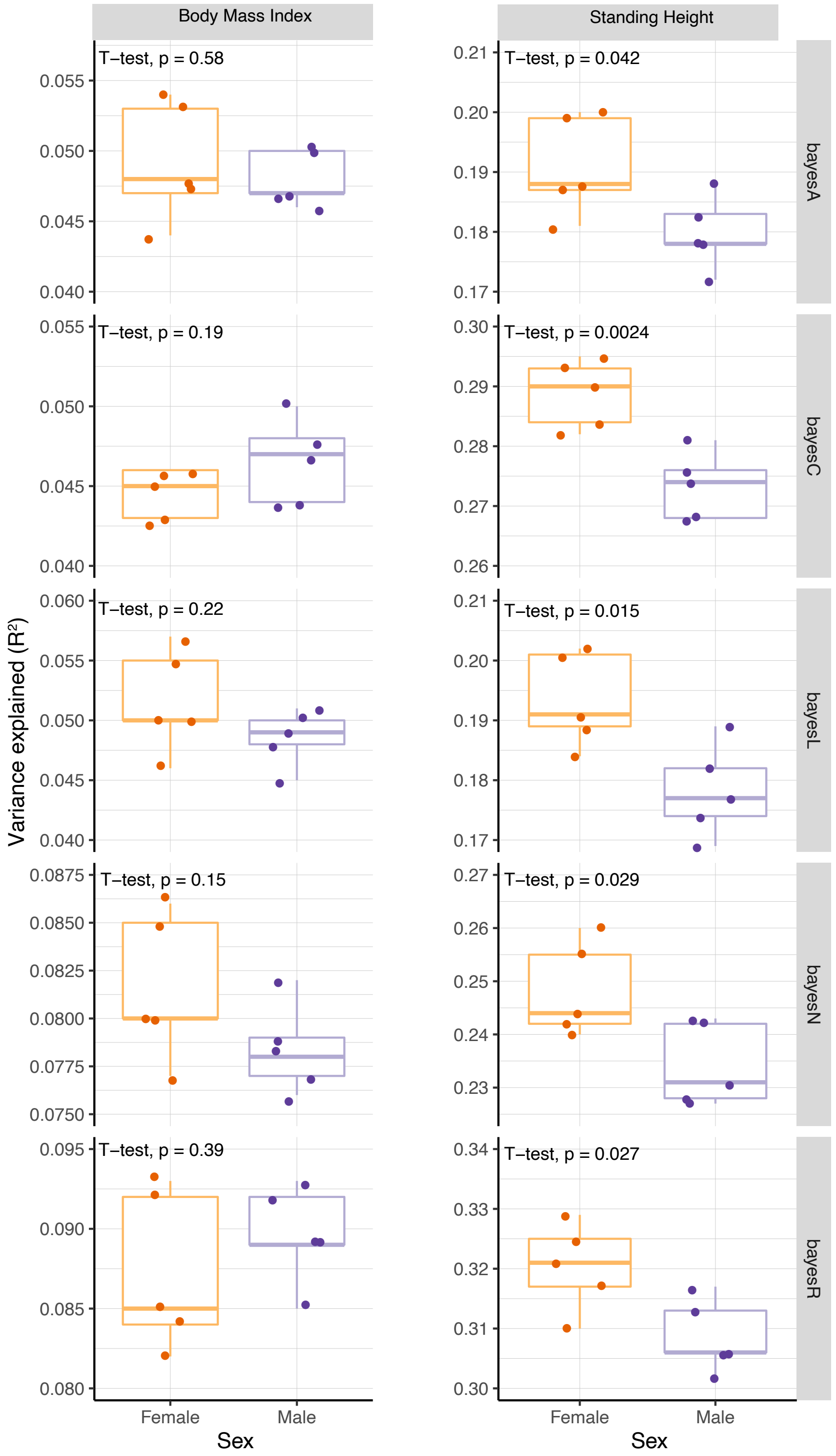

Figure S2: Boxplot of predictive performances (quantified as variance explained, R^2) for body mass index and standing height within the UK Biobank across the five training/validation cohorts. The T-test P-values is testing differences in mean predictive performance between females and males.

increased the prediction accuracy for BMI for males and females with >20% (Figure S3), despite the low genetic correlation between height and BMI (Figure S3). Similarly, leveraging the genomic pleiotropy between BMI and height for males and females separately, increased the prediction accuracy for standing height by almost 15% (Figure S4). Applying the selection index MT methodology to the BLR models,

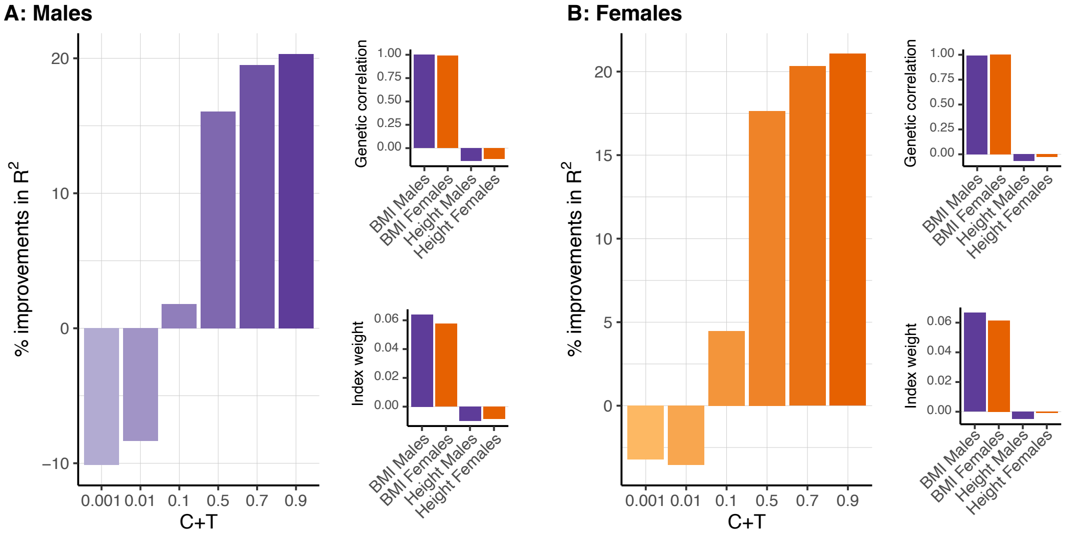

Figure S3: Improvements in predictive performance for body mass index (BMI) for A: males and B: females, when leveraging the genetic correlation between BMI and standing height for males and females separately.

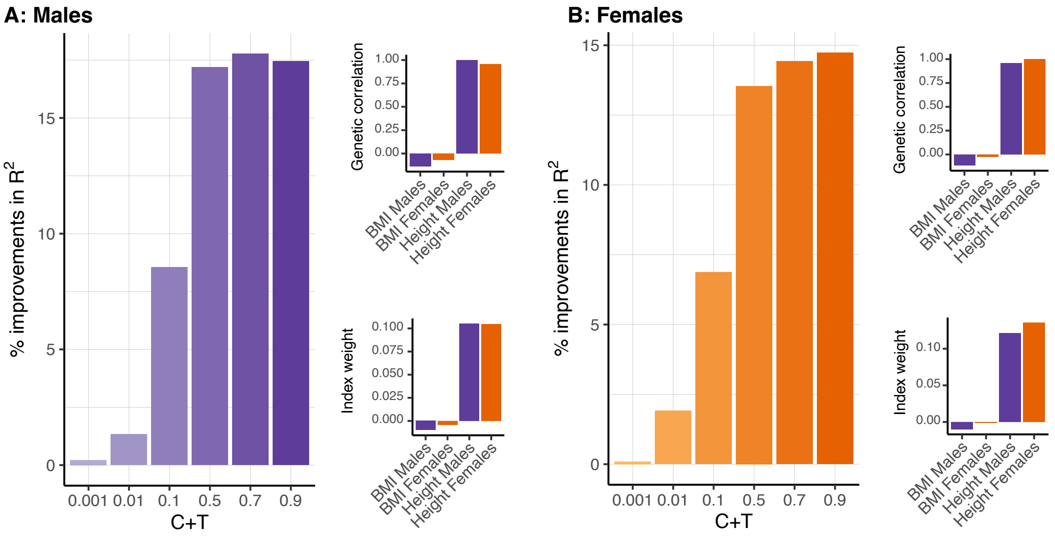

Figure S4: Improvements in predictive performance for standing height for A: males and B: females, when leveraging the genetic correlation (estimated with LDSC) between BMI and standing height for males and females separately.

further improved the predictive accuracy for all five BLR models with 20-60% for BMI and 10-45% for standing height (Figure S5A). Interestingly, MT-PGS based on BayesR displayed same amount of prediction accuracy as single trait GS with BayesR when the summary statistics are trained on both males and females (Figure S5B), i.e., approximately double the sample size to the analysis of males and females separately.

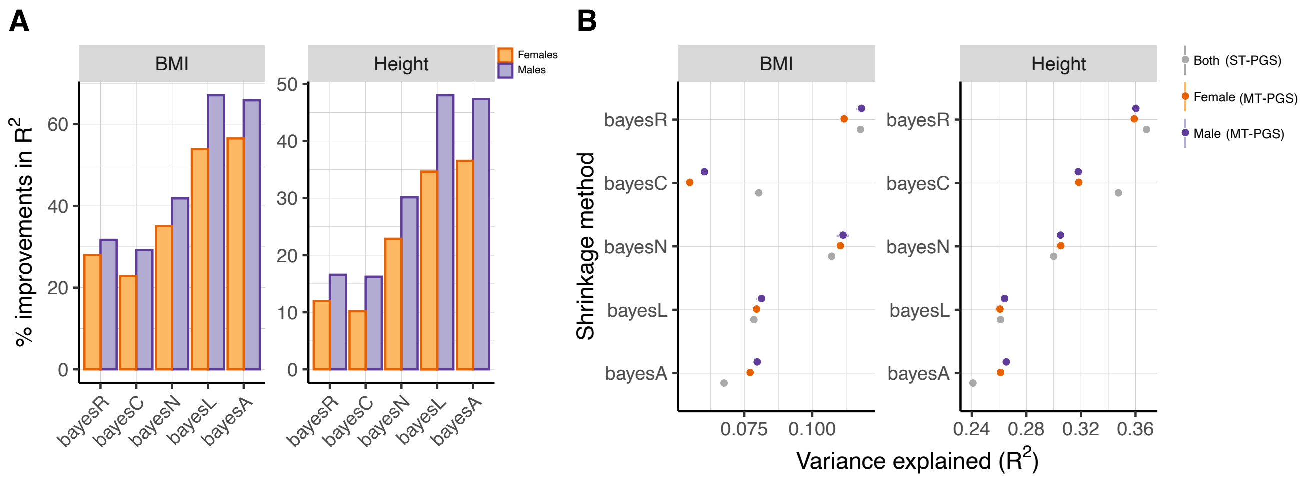

Figure S5: Average prediction accuracies of the five different Bayesian Linear Regression (BLR) models (error bars are standard errors of the mean) for body mass index (BMI) and standing height for A) males and females separately using the multiple-trait approach (MT-PGS), and for B) both sexes combined using the single-trait approach (ST-PGS) quantified as variance explained (R^2).

S4.2 Estimated genetic parameters

Computing the index weights used in constructing the MT-PGS requires estimates of trait heritability ($\hat{h}^2$) and the genetic correlation ($\hat{r}_g$) among the phenotypes which can, for example, be achieved using summary statistics-based LD score regression (as implemented in qgg). However, our implementation of the different BLR models also provide accurate estimates of the heritability. For example, using BayesC, we estimated the heritability for BMI and standing height for each sex separately and the two sexes combined. The heritability for height and BMI were within trait the same across sexes and combined with very small differences in estimates across the five training sets (Figure S6A).

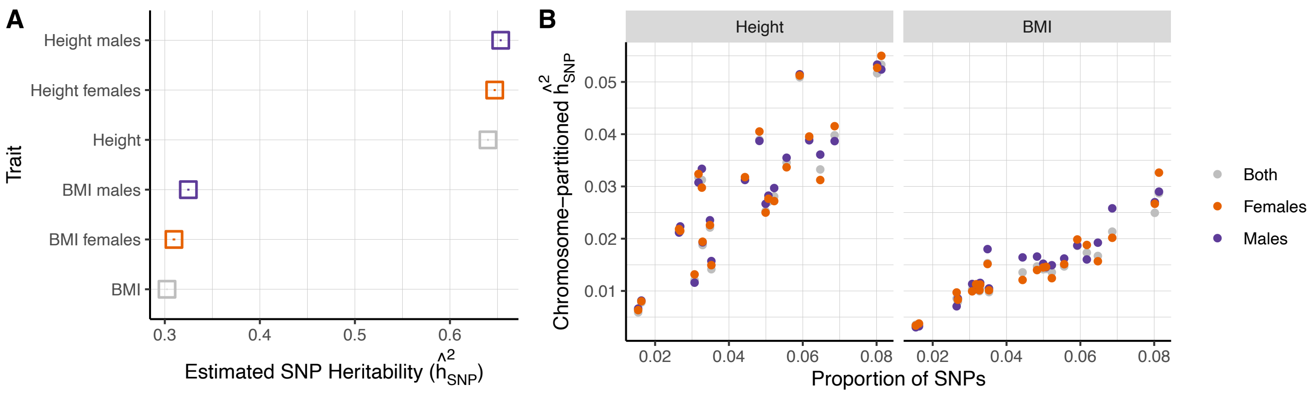

Figure S6: Bayesian linear regression (BLR) model with bayesR shrinkage used to compute A) SNP heritability estimation (h^2_{SNP}) for standing height and body mass index (BMI) for each sex separately and together, and B) partitioned heritability into autosomal chromosomes for height and BMI. For both panels it is the mean heritability across five training sets that is shown.

Furthermore, we partitioned the heritability across different marker sets, e.g., by those on each autosomal chromosome (Figure S6B). Finally, we partitioned the genetic correlation, which showed varying degrees of positive genetic correlation across the 22 autosomes for standing height between sexes (Figure S7). For BMI the pattern was different with almost the same level of genetic correlation among chromosomes (Figure S7), and the genetic correlation between BMI and height between sexes showed different

patterns across autosomes.

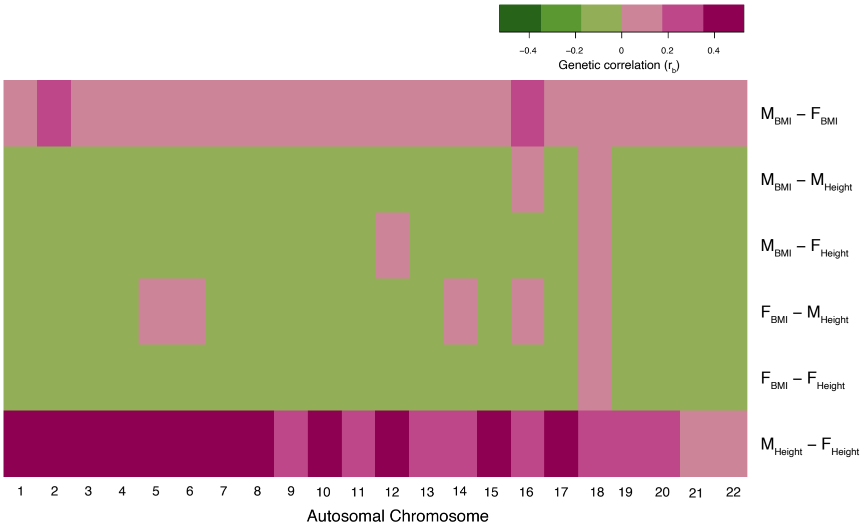

Figure S7: Partitioned genetic correlation (here approximated as the correlation among BayesC estimated marker effects, r_b) among autosomal chromosomes for standing height and body mass index (BMI) for males and females.

Besides estimating how much of the phenotypic variance we can explain with common genetic variants, or how large a fraction of the genetic contribution that is shared across traits, it can also be useful to estimate the proportion of variants with a likely causal effect. As example, using BayesR, we estimated the proportion of variants with no, small, moderate, or large effect for the two traits. BMI and height have to a large extent a similar genetic profile with one big difference, namely that BMI has <5% variants with large effects, while standing height has approximately 15% variants with an estimated large effect (Figure S8).

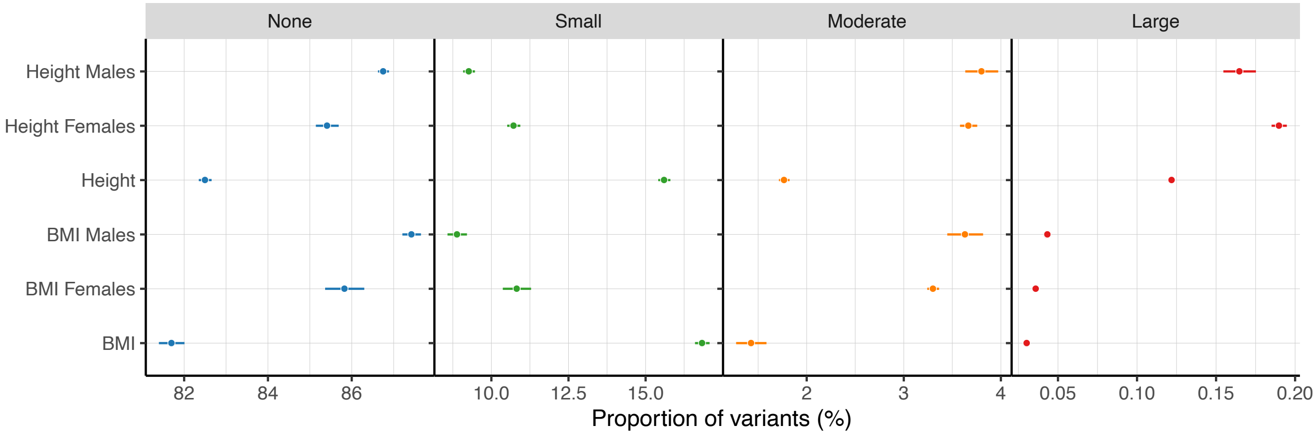

Figure S8: Proportion of genetic variants with no, small, moderate, or large effect sizes, estimated with BayesR. Each point is the mean across the five training sets, and error bars represents the standard error of the mean.

S4.3 Bayesian fine-mapping

BLR models account for the underlying genetic architecture by sampling marker variances from different prior distributions and by accounting for linkage disequilibrium between markers and thus proposed to have

Gene set (total set)	Height		BMI	
	Males	Females	Males	Females
Sequence ontology (7)	5	5	0	1
Genes (24,059)	2	3	5	1
Gene ontologies (4544)	13	13	0	2
Pathways (2243)	0	1	4	0
Protein complexes (4948)	0	1	1	0
Chemical complexes (94,884)	0	0	0	0

Table S5: Summary of number of marker sets within the different categories with an false discovery rate (FDR) corrected P-value <0.05.

greater power to detect causal associations (Lloyd-Jones *et al.*, 2019). As an example, we used BayesC to fine-map genetic markers for standing height, focusing on chromosome 1. As expected, because of the BayesC prior shrinkage, most of the markers on chromosome 1 received a zero-effect size (Figure S9A). We computed the T_{PIP} for a sliding window of 100 markers to make inference on the presence of causal markers in these windows. In particular, one region on chromosome 1 showed high T_{PIP} compared to the other regions (Figure S9B). This region contains the *PAPPA2* gene which is one of the many known QTLs for standing height (Figure S9C) (Allen *et al.*, 2010; Kichaev *et al.*, 2019; He *et al.*, 2015). Likewise, zooming in on another region with lower T_{PIP} than the *PAPPA2*-region, we find other regions with high posterior inclusion probabilities, such as the intergenic region between the genes *H6PD* and *SPSB1*, which are known genes for height (Berndt *et al.*, 2013; Sakaue *et al.*, 2021; Wood *et al.*, 2014).

S4.4 Bayesian-based gene set enrichment analysis

An overview of the number of identified genetic marker sets with a false discovery rate below 5% is shown in Table S5. A few examples of association are given below, while a complete overview can be found in Supplementary Table S2 and at our accompanied homepage (https://qganalytics.com/gact). We identified *ZNF385C* and *CDIP1* as being associated with standing height for both males and females, while *ANKS6* was female-specific for height. For both sexes and traits, we show an enrichment in the sequence ontology 'coding region'. For gene ontologies we identified a total of 13 terms that were associated with height in both sexes, including terms within the molecular function for 'negative regulation of transcription by RNA polymerase II' (GO:0000122), 'positive regulation of DNA-templated transcription' (GO:0045893), 'DNA-binding transcription factor activity and RNA polymerase II-specific' (GO:0000981). Only a few Reactome pathways were identified, in particular DNA synthesis (R-HSA-164516 and R-HSA-164525) for BMI for males. None of the chemical complexes survived correction for multiple testing.

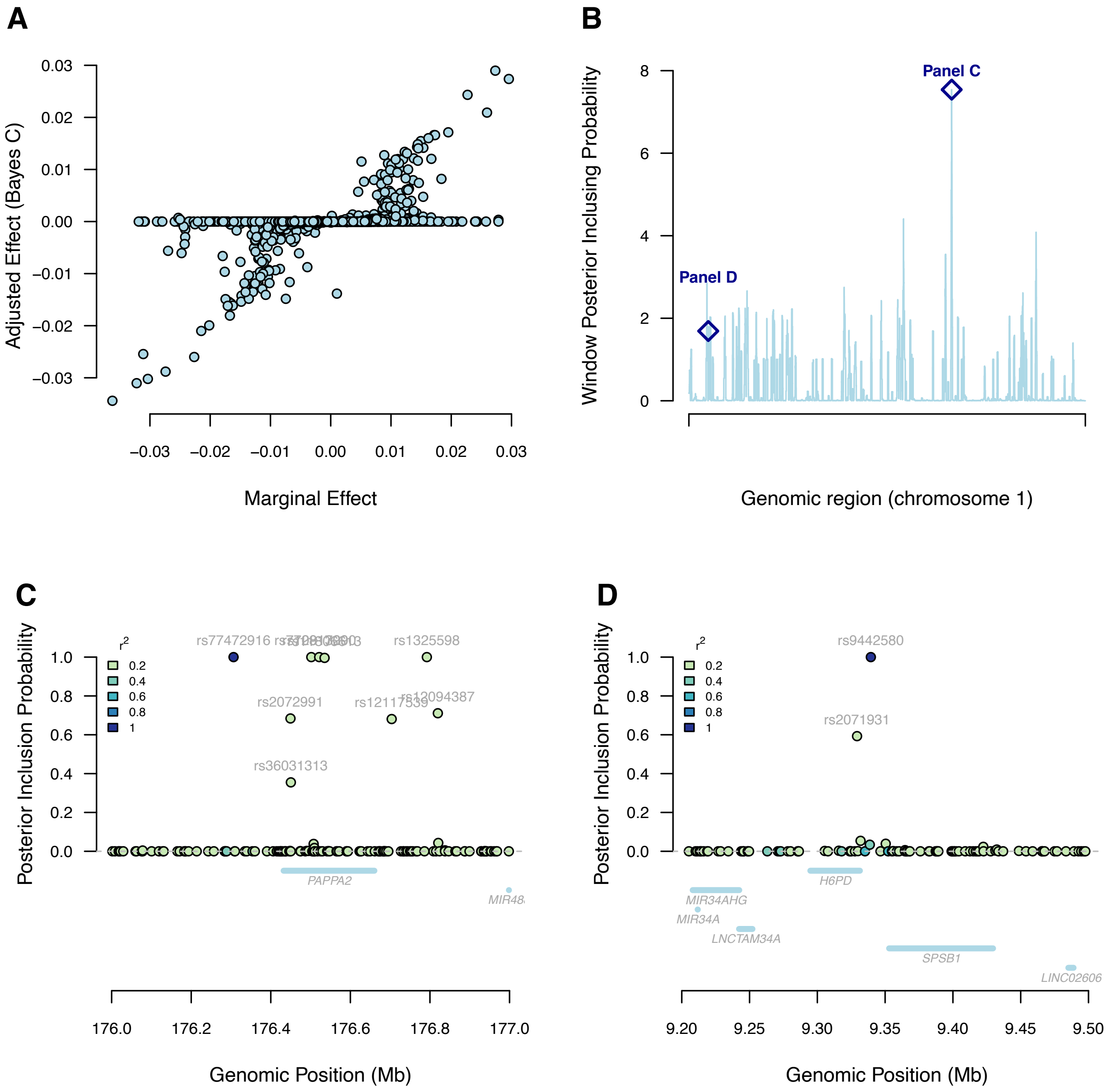

Figure S9: Example of genetic fine mapping using a Bayesian linear regression (BLR) model with Bayes C shrinkage for standing height on chromosome 1. **A**) Comparison of ordinary linear squares (OLS) marginal SNP effects to adjusted SNP-effects after bayesC adjustment. **B**) Chromosome 1 Manhattan-like plot of windows of posterior inclusion probabilities (bayesC, sliding window size of 100 SNPs) for standing height. **C-D**) Locus zoom on two genomic regions on chromosome 1 (see diamond indicators in panel **B**) of individual variants' posterior inclusion probability. Color grading indicates degree of linkage disequilibrium (LD) measured by Pearson's correlation (r^2). Blue segments below points indicate gene transcription start and stop sites (UCSC, GRCh73).

References

Allen, H. L., *et al.* (2010) Hundreds of variants clustered in genomic loci and biological pathways affect human height. *Nature*, 467, 832-838.

Ashburner, M., *et al.* (2000) Gene Ontology: tool for the unification of biology The Gene Ontology Consortium. *Nat Genet*, 25, 25-29.

Berndt, S. I., *et al.* (2013) Genome-wide meta-analysis identifies 11 new loci for anthropometric traits and provides insights into genetic architecture. *Nat Genet*, 45, 501-512.

Bycroft, C., *et al* (2018) The UK Biobank resource with deep phenotyping and genomic data. *Nature*, 562, 203-209.

Chang, C. C., *et al.* (2015). Second-generation PLINK: Rising to the challenge of larger and richer datasets. *Gigascience*, 4, 1-16.

Cheng, H., *et al.* (2018). Genomic prediction from multiple-trait bayesian regression methods using mixture priors. *Genetics*, 209, 89-103.

Carlson, M. (2019) `org.Hs.eg.db`: Genome wide annotation for Human.

Choi, S.W., *et al.* (2020) Tutorial: a guide to performing polygenic risk score analyses. *Nat Protoc*, 15, 2759-2772.

Dudbridge, F. (2013) Power and Predictive Accuracy of Polygenic Risk Scores. *PLoS Genet*, 9.

Erbe, M., *et al* (2012) Improving accuracy of genomic predictions within and between dairy cattle breeds with imputed high-density single nucleotide polymorphism panels. *J Dairy Sci*, 95, 4114-4129.

Euesden, J., *et al.* (2015) PRSice: Polygenic Risk Score software. *Bioinformatics*, 31, 1466-1468.

Gianola, D., *et al.* (2009) Additive genetic variability and the Bayesian alphabet. *Genetics*, 183, 347-363.

Gianola, D. (2013) Priors in whole-genome regression: The Bayesian alphabet returns. *Genetics*, 194, 573-596.

Gillespie, M., et al. (2022) The reactome pathway knowledgebase 2022. *Nucleic Acids Res*, 50, D687-D692.

Habier, D., *et al.* (2011) Extension of the bayesian alphabet for genomic selection. *BMC Bioinformatics*, 12.

He, M., *et al.* (2015) Meta-analysis of genome-wide association studies of adult height in East Asians identifies 17 novel loci. *Hum Mol Genet*, 24, 1791-1800.

Jia, Yi and Jannink, Jean-Luc. (2012) Multiple-trait genomic selection methods increase genetic value prediction accuracy. *Genetics*, 192, 1513-1522.

Kichaev, G., *et al.* (2019) Leveraging polygenic functional enrichment to improve GWAS power. *Am J Hum Genet*, 104, 65-75.

Legarra, A., *et al.* (2011) Improved Lasso for genomic selection. *Genet Res*, 93, 77-87.

de Leeuw, C. A., *et al.* (2016) The statistical properties of gene-set analysis. *Nat Rev Genet*, 17, 353-364.

Loh, P. R., *et al.* (2015) Efficient Bayesian mixed-model analysis increases association power in large cohorts. *Nat Genet*, 47, 284-290.

de Los Campos, G., *et al.* (2009) Predicting quantitative traits with regression models for dense molecular markers and pedigree. *Genetics*, 182, 375-385.

Lloyd-Jones, L. R., *et al.* (2019) Improved polygenic prediction by Bayesian multiple regression on summary statistics. *Nat Commun*, 10, 1-11.

Maier, R. M., *et al.* (2018) Improving genetic prediction by leveraging genetic correlations among human diseases and traits. *Nat Commun*, 9, 1-17.

Manolio, T. A., *et al.* (2009) Finding the missing heritability of complex diseases. *Nature*, 461, 747-753.

Marees, A. T., *et al.* (2018) A tutorial on conducting genome-wide association studies: Quality control and statistical analysis. *Int J Methods Psychiatr Res*, 27, 1-10.

Mbatchou, J., *et al.* (2021) Computationally efficient whole-genome regression for quantitative and binary traits. *Nat Genet*, 53, 1097-1103.

Meuwissen, T. H. E., *et al.* (2001) Prediction of total genetic value using genome-wide dense marker maps. *Genetics*, 157, 1819-1829.

Moser, G., *et al.* (2015) Simultaneous discovery, estimation and prediction analysis of complex traits using a Bayesian mixture model. textitPLoS Genet, 11, 1-22.

Park, T. and Casella, G. (2008) The Bayesian Lasso. *J Am Stat Assoc*, 103, 681-686.

Patxot, M.,*et al.* (2021). Probabilistic inference of the genetic architecture underlying functional enrichment of complex traits. *Nat Commun*, 12, 6972.

Pérez, P., *et al.* (2010) Genomic-enabled prediction based on molecular markers and pedigree using the bayesian linear regression package in R. *Plant Genome*, 3.

Privé, F., *et al.* (2019) Making the most of clumping and thresholding for polygenic scores. *Am J Hum Genet*, 105, 1213-1221.

Privé, F., *et al.* (2021) LDpred2: better, faster, stronger. *Bioinformatics*, 36, 5424-5431.

Purcell, S. M., *et al.* (2009) Common polygenic variation contributes to risk of schizophrenia and bipolar disorder. *Nature*, 460, 748-752.

Rohde, P. D., *et al.* (2016) Covariance Association Test (CVAT) identifies genetic markers associated with schizophrenia in functionally associated biological processes. *Genetics*, 203, 1901-1913.

Rohde, P. D., *et al.* (2020) qgg: An R package for large-scale quantitative genetic analyses. *Bioinformatics*, 36, 2614-2615.

Rohde, P. D., *et al.* (2021) Multi-trait genomic risk stratification for type 2 diabetes. *Front Med*, 8.

Sakaue, S., *et al.* (2021) A cross-population atlas of genetic associations for 220 human phenotypes. *Nat Genet*, 53, 1415-1424.

Schaid, D . J., *et al.* (2018) From genome-wide associations to candidate causal variants by statistical fine-mapping. *Nat Rev Genet*, 19, 491-504.

Sørensen, I. F., *et al.* (2017) Multiple trait covariance association test identifies gene ontology categories associated with chill coma recovery time in *Drosophila melanogaster*. *Sci Rep*, 7, 2413.

Szklarczyk, D., *et al.* (2016) STITCH 5: Augmenting protein-chemical interaction networks with tissue and affinity data. *Nucleic Acids Res*, 44, D380-D384.

Szklarczyk, D., *et al.* (2019) STRING v11: Protein-protein association networks with increased coverage, supporting functional discovery in genome-wide experimental datasets. *Nucleic Acids Res*, 47, D607-D613.

Vilhjálmsson, B. J., *et al.* (2015) Modeling linkage disequilibrium increases accuracy of polygenic risk scores. *Am J Hum Genet*, 97, 576-592.

Wood, A. R., *et al.* (2014) Defining the role of common variation in the genomic and biological architecture of adult human height. *Nat Genet*, 46, 1173-1186.

Wu, M. C., *et al.* (2011) Rare-variant association testing for sequencing data with the Sequence Kernel Association Test. *Am J Hum Genet*, 89, 82-93.

Timpson, N. J. *et al.* (2018) Genetic architecture: The shape of the genetic contribution to human traits and disease. *Nat Rev Genet*, 19, 110-124.

Yang, J., *et al.* (2011) Genomic inflation factors under polygenic inheritance. *Eur J Hum Genet* 19, 807-812.

Yu, J., *et al.* (2006) A unified mixed-model method for association mapping that accounts for multiple levels of relatedness. *Nat Genet*, 38, 203-208.

Zhou, X. and Stephens, M. (2012) Genome-wide efficient mixed-model analysis for association studies. *Nat Genet*, 44, 821-824.
